# A data-driven evaluation of a train-the-trainer program for delirium management in ICUs: a mixed-methods study integrating multidimensional outcome metrics and qualitative insights

**DOI:** 10.3389/fneur.2026.1824373

**Published:** 2026-06-01

**Authors:** Yadi Feng, Yuan Yuan, Ran Zhang, Xiaoping Yi, Yana Xing, Weixin Cai

**Affiliations:** Beijing Tiantan Hospital, Capital Medical University, Beijing, China

**Keywords:** data driven evaluation, delirium, implementation science, intensive care units, Kirkpatrick model, mixed methods, nursing education, social cognitive theory

## Abstract

**Background:**

Delirium remains a prevalent and serious complication in intensive care units (ICUs), yet the translation of evidence-based monitoring and management into routine nursing practice faces persistent implementation gaps. Sustainable and scalable educational strategies are urgently needed to address this challenge.

**Objectives:**

This study employed a mixed-methods approach to comprehensively evaluate the impact of a train-the-trainer (TTT) delirium education program using a multidimensional outcome framework. Quantitatively, we assessed changes in ICU nurses’ competencies across the learning, behavior, and results levels of the Kirkpatrick model. Qualitatively, we explored the underlying experiential mechanisms and contextual drivers to explain the quantitative outcomes and inform program optimization.

**Methods:**

A mixed-methods quasi-experimental study with an embedded qualitative component was conducted in a tertiary ICU in China. Forty-seven nurses participated in the TTT program. Quantitative data acquisition included validated instruments for delirium knowledge assessment, standardized clinical simulation evaluations using the CAM-ICU tool, and the General Self-Efficacy Scale, collected at baseline and three months post-intervention. For qualitative data acquisition, semi-structured interviews were conducted with 12 purposively sampled nurses to explore experiential learning trajectories, behavioral drivers, and implementation barriers. Thematic analysis was guided by Social Cognitive Theory and performed using systematic coding procedures.

**Results:**

Quantitative analysis revealed statistically significant improvements across all outcome metrics: delirium knowledge scores (*p* < 0.05), clinical assessment accuracy (*p* < 0.05), and self-efficacy (*p* < 0.001). Qualitative analysis identified four core thematic patterns: (1) Experiential learning trilogy—simulation, practice, and teaching; (2) From experience to evidence—the construction of clinical confidence; (3) Environmental forces—barriers and facilitators in practice implementation; and (4) Systemic spillover effects—enhanced interprofessional communication and recognition of hypoactive delirium. Nurses also provided actionable recommendations for training optimization, notably the need for standardized video resources to ensure content consistency.

**Conclusion:**

The TTT program significantly enhanced ICU nurses’ delirium care competencies, driven by simulation-based practice, repeated application, and peer teaching. Contextual challenges and the need for standardized video resources inform future implementation and fidelity considerations. This study provides a feasible, scalable framework for evidence-based delirium care.

## Highlights

- Delirium is a major concern in ICUs, and accurate assessment requires formal, sustained training.
- Multidimensional Outcome Assessment: The TTT program demonstrated statistically significant improvements across all Kirkpatrick levels: knowledge acquisition (learning), clinical simulation accuracy (behavior), and self-efficacy (results), providing comprehensive evidence of program effectiveness.
- Qualitative Mechanism Discovery: Thematic analysis revealed that simulation-based experiential learning, repeated practice opportunities, and peer teaching constitute the core mechanisms driving confidence construction and competence development.
- Contextual Implementation Barriers: Environmental factors—particularly patient non-cooperation and challenges in assessing neurologically compromised patients—highlight the limitations of current assessment tools in specialized clinical settings and inform future tool refinement.
- Program Optimization Insights: Nurses’ identification of the need for standardized video resources suggests that integrating blended learning approaches with the TTT model could enhance training fidelity and scalability across diverse ICU contexts.
- Methodological Integration: The mixed-methods design provides a holistic understanding of both program effectiveness and underlying mechanisms, demonstrating the value of integrating quantitative outcome metrics with qualitative experiential data in healthcare education research.

## 1 Introduction

Delirium is an acute disturbance of attention and cognitive function and is one of the most common complications in patients in the intensive care unit (ICU). Clinically, delirium that occurs in ICU patients is often referred to as ICU delirium. Depending on the study population, it has an incidence ranging from 33.87 to 81.7% (1–3). The occurrence of delirium increases both the morbidity and mortality rates of patients (4), prolongs hospitalization and recovery time, and affects patients’ prognosis (5, 6). Standardized monitoring and management of delirium are integral components of critical care guidelines.

The American College of Critical Care Medicine recommends the routine use of the Confusion Assessment Method for the Intensive Care Unit (CAM-ICU) to assess delirium in critically ill adult patients (7). Although the CAM-ICU has been validated by experts, trained ICU nurses, physicians, and researchers and has shown high sensitivity and specificity, in ICU practice, the sensitivity of the CAM-ICU when used by nurses decreases to 47% (8). The main reasons for this are related primarily to ICU nurses’ lack of education in delirium assessment, perceptions of assessment tools as complex, insufficient experience using delirium assessment tools, and low confidence (9, 10). Therefore, there is an urgent need to identify effective ways to address the challenges of delirium management in ICU nursing practice.

In recent years, a variety of training methods, including lectures, videos, and web-based courses, have been employed to improve ICU nurses’ delirium assessment skills (6, 11). Nonetheless, gaps persist. The limited number of ICU nurses, the complexity of shift work, and the intricacy of delirium assessment hinder the integration of training into practice. Even with remote (12) and case-based training methods (11), nurses still make inaccurate assessments and find the tools complex (8–10). Training lacks practical guidance, is delivered in a one-to-many format, and has poor coverage due to ICU shift schedules, which diminishes its effectiveness.

The Train-the-Trainer (TTT) program is innovative. It is a structured initiative designed to enhance learning in healthcare settings (13). It employs trainers who, after receiving training, conduct subsequent training in a pyramid-like manner. This approach enables more nurses to learn about delirium, addressing the shortage of trained personnel.

In the present study, we examined the effects of the TTT delirium training program on ICU nurses’ knowledge and assessment accuracy of delirium. As a cost-effective model, previous research indicates that the TTT program outperforms traditional lecture-based training, making it an ideal approach for enhancing ICU nurses’ delirium knowledge and assessment accuracy, which is precisely the focus of our current research (6). To gain a comprehensive understanding of both the outcomes and the underlying processes of this educational intervention, this study employed a mixed-methods design, combining quantitative evaluation with in-depth qualitative exploration.

## 2 Methods

### 2.1 Study design and data architecture

This study utilized an explanatory sequential mixed-methods design underpinned by a data-driven analytical framework. The quantitative phase implemented a quasi-experimental pre-post design to evaluate multidimensional outcomes. In contrast, the qualitative phase applied thematic analysis to discern experiential patterns and elucidate the quantitative results.

### 2.2 Study cohort and data source

The study cohort was derived from the intensive care unit (ICU) of a tertiary Grade A hospital in Beijing, forming a high-quality clinical nursing dataset. A total of 47 ICU nurses were enrolled in the quantitative data acquisition phase, all actively employed at the study hospital. To ensure data integrity and minimize confounding variables, exclusion criteria eliminated nurses pursuing further education, nursing interns, those on long-term leave, or studying abroad. For the qualitative data acquisition, a purposive sampling strategy was employed to select 12 nurses from the original cohort, ensuring diversity across key demographic and professional dimensions including age (range: 26–40 years), clinical title, and ICU experience (range: 3–15 years). Sample size was determined by thematic saturation—the point at which additional interviews yielded no novel informational patterns (14).

### 2.3 Ethical compliance and data privacy protocols

This study received ethical approval from the Institutional Review Board (IRB) of Beijing Tiantan Hospital, Capital Medical University (Approval No. KY2024-300-02). Written informed consent was obtained from all participating nurses prior to data acquisition. All personally identifiable information was anonymized through a systematic de-identification protocol, and data were stored in encrypted, access-controlled repositories to ensure confidentiality and regulatory compliance. Separate ethical approval and informed consent, including provisions for audio recording and use of anonymized quotations, were obtained for the qualitative data collection component.

### 2.4 Intervention system: a multi-phase TTT-based training architecture

#### 2.4.1 System overview

The intervention was designed as a structured, multi-phase Train-the-Trainer (TTT) program, implemented under the supervision of the ICU head nurse, who served as the principal training facilitator. The facilitator held qualifications as a clinical educator and had accumulated 8 years of specialized research experience in delirium management.

#### 2.4.2 Phase 1: trainer candidate selection and preparation

Three senior ICU nurses were selected as trainer candidates based on predefined inclusion criteria: ① ≥ 5 years of ICU clinical experience; ② bachelor’s degree or higher; ③ teaching qualifications; and ④ demonstrated language proficiency and organizational capacity. The demographic profile of the selected trainers showed a mean age of 33.67 ± 3.30 years, with two holding bachelor’s degrees and one holding a master’s degree.

The preparation protocol comprised two components:

1. Expert-Validated Content Development: Training materials were developed through systematic literature review and validated by a panel of seven experts, including a nursing education specialist, a critical care medicine specialist, a critical care nursing specialist, and four senior ICU nurses with over a decade of experience in delirium patient management. Expert selection criteria included: (1) senior professional title (associate senior or above); (2) substantial clinical or educational experience in delirium care, critical care, or nursing education; and (3) willingness to participate and provide informed recommendations. Expert feedback was systematically integrated to optimize content validity and clinical relevance.
2. Simulation-Based Assessment Training: Trainer candidates underwent practical assessment training using standardized patient cases. Case scenarios, adapted from established research protocols, incorporated detailed medical histories, current conditions, behavioral patterns, and clinical triggers. Standardized patients received role-based training and were placed in realistic ICU environments equipped with monitoring devices and medical equipment to ensure ecological validity of the assessment scenarios.

#### 2.4.3 Phase 2: trainer competency validation

During this phase, the three trainer candidates delivered the training protocol to each other in a structured 90-min session comprising: a 20-min didactic lecture, a 60-min case assessment practicum using standardized patients, and a 10-min interactive Q&A session. Following delivery, the trainers underwent competency assessment by the principal facilitator to validate their readiness for departmental implementation.

#### 2.4.4 Phase 3: departmental implementation

Over a one-month period, the validated trainers delivered the identical 90-min training protocol to the 47 departmental nursing staff. Sessions were scheduled three times weekly to accommodate ICU shift patterns. Upon completion, all 47 nursing staff members underwent standardized competency assessment conducted by the trainers.

### 2.5 Outcome measures and evaluation metrics

Training effectiveness was evaluated using a multidimensional outcome assessment framework based on the Kirkpatrick model, operationalized across three hierarchical levels: learning acquisition, behavioral application, and self-efficacy outcomes.

#### 2.5.1 Learning level: knowledge acquisition metrics

A 27-item delirium knowledge assessment instrument was developed through systematic literature review, encompassing six domains: basic concepts (5 items), clinical manifestations (4 items), hazards (5 items), risk factors (3 items), prevention and management measures (5 items), and tool application (5 items). The instrument employed binary scoring (correct = 1, incorrect = 0), yielding a total score range of 0–27. Psychometric properties were evaluated through:

1. Difficulty Index (P): Calculated as P = R/N, where R represents the number of correct responses and N represents the total number of respondents.
2. Discrimination Index: This was assessed using Spearman’s rank correlation analysis, which compared individual item scores with total scores. Values of ≥0.2 were considered indicative of acceptable discriminatory power. Internal consistency reliability was evaluated through Cronbach’s alpha coefficient, which produced a value of 0.616, reflecting acceptable reliability for this study. This moderate value is primarily attributed to the small sample size (*N* = 47) and the multidimensional characteristics of the delirium knowledge domains, rather than to insufficient item quality.

#### 2.5.2 Behavior level: clinical assessment accuracy

Assessment accuracy was evaluated using standardized patient cases and the CAM-ICU delirium assessment protocol. The protocol comprised two stages: indication for delirium assessment and confirmation of delirium presence, with evaluation of four characteristic features: acute changes or fluctuations in mental status, inattention, altered level of consciousness, and disorganized thinking (Table 1).

**Table 1:** Difficulty value and discrimination of each topic of the test paper.

Domain	Item no.	Difficulty index	Discrimination index
Basic concepts	1	0.90	0.11
Basic concepts	2	0.89	0.40
Basic concepts	5	0.15	0.25
Basic concepts	6	0.60	0.23
Basic concepts	24	0.40	0.27
Clinical manifestations	7	0.15	0.08
Clinical manifestations	8	0.87	0.58
Clinical manifestations	9	0.92	0.15
Clinical manifestations	10	0.62	0.53
Risks	11	0.29	0.48
Risks	12	0.42	0.42
Risks	13	0.89	0.50
Risks	14	0.45	0.33
Risks	15	0.81	0.44
Risk factors	3	0.58	0.24
Risk factors	16	0.77	0.45
Risk factors	17	0.85	0.32
Prevention and management	4	0.98	0.18
Prevention and management	18	0.43	0.37
Prevention and management	19	0.79	0.47
Prevention and management	20	0.83	0.53
Prevention and management	21	0.89	0.32
Tool application	22	0.11	0.35
Tool application	23	0.26	0.58
Tool application	25	0.30	0.49
Tool application	26	0.09	0.27
Tool application	27	0.60	0.24

Each case was assessed from 0 to 10, with 2 points for the first stage indication assessment and 8 points for the four second stage CAM-ICU features with each contributing 2 points. The total score (summed sum of five cases) ranged from 0 to 50, higher scores indicated more accurate assessment. Content validity was verified by independent review by seven domain experts with CVI of 0.89. Expert panel age: 42.86 ± 4.85 years and average domain experience: 20.00 ± 6.68 years. Professional backgrounds of the experts can be found in Table 2.

**Table 2:** Demographic and professional information of the experts.

Project	Category	Number of cases	Proportion
Domain of expertise	Psychiatric specialist	1	14.29%
	Intensive care medicine specialist	1	14.29%
	Nursing education specialist	1	14.29%
	Intensive care nursing specialist	4	57.14%
Professional title	Senior title	5	71.43%
	Intermediate title	2	28.57%
Education level	Bachelor’s degree	3	42.86%
	Master’s degree	1	14.29%
	Doctoral degree	3	42.86%

Experts may contribute to various domains; for instance, an individual could serve as both an Intensive care nursing specialist and hold a senior title. The figures in each subcategory indicate the number of experts who identified with that particular subcategory; totals may exceed *N* due to multiple selections.

#### 2.5.3 Results level: self-efficacy measurement

Self-efficacy was assessed using the Chinese version of the General Self-Efficacy Scale (GSES), adapted by Zhang and Schwarzer. The 10-item instrument employs a 4-point Likert scale (4 = completely correct, 3 = mostly correct, 2 = somewhat correct, 1 = completely incorrect). Total scores were categorized as: <10 (very low self-efficacy), 11–20 (low self-efficacy), 21–30 (moderate self-efficacy), and 31–40 (very high self-efficacy).

### 2.6 Data acquisition protocol

#### 2.6.1 Quantitative data acquisition

Delirium knowledge assessments and GSES measurements were administered to all 47 ICU nurses at two time points: baseline (pre-training) and three months post-training. Assessments were conducted during centralized sessions with standardized administration protocols to minimize measurement bias. A total of 94 questionnaires were distributed, achieving a 100% effective response rate and complete data capture.

Delirium assessment accuracy data were obtained by standard on-site assessments carried out in baseline (pre-training) and 3 months post-training. All 47 nurses completed assessments, resulting in 100% data capture.

#### 2.6.2 Qualitative data acquisition

Semi-structured interviews were conducted three months post-training, each lasting 30–45 min. An interview guide, informed by Social Cognitive Theory and aligned with Kirkpatrick evaluation levels, explored: (a) learning experiences within the TTT framework; (b) perceived changes in knowledge, behavior, and confidence; (c) facilitators and barriers to practice implementation; (d) perceptions of the peer-teaching model; and (e) recommendations for training optimization. All interviews were audio-recorded, transcribed verbatim, and de-identified for analysis. Thematic saturation was assessed sequentially: every 3 interviews, we conducted a preliminary thematic analysis. Saturation was reached if two interviews in succession (9th-10th and 11th-12th) did not yield new codes or themes. Final 2 interviews showed no additional insights, saturation was reached with 12 participants.

### 2.7 Analytical framework

#### 2.7.1 Quantitative data analysis

Statistical analyses were performed using SPSS (Version 17.0). Descriptive statistics were computed for all variables: categorical data were expressed as frequencies and percentages; normally distributed continuous data were expressed as means ± standard deviations; non-normally distributed continuous data were expressed as medians with interquartile ranges. For comparative analysis of pre-training and three-month post-training outcomes, paired-sample Wilcoxon signed-rank tests were applied to non-normally distributed continuous data. Statistical significance was defined as *p* < 0.05. Effect sizes for Wilcoxon signed-rank tests were calculated as *r* = Z/√N, where *N* = 47. Before comparing, all continuous variables were normalized by Shapiro–Wilk test. Variables not normalized were not evaluated by nonparametric methods.

#### 2.7.2 Qualitative data analysis

Interview transcripts were analyzed using thematic analysis following the Braun and Clarke six-phase framework (15): familiarization, initial code generation, theme search, theme review, theme definition and naming, and report production. Two researchers conducted independent coding to enhance analytical reliability. NVivo 12 software was utilized for data management and code organization. Methodological rigor was ensured through peer debriefing, reflexive journaling, and systematic presentation of illustrative quotations (16).

## 3 Results

### 3.1 Learning-level evaluation results

Of the 47 ICU nurses, the majority were female (78.7%). Most were in the 26–30 age group (38.3%). A significant majority (89.4%) held a bachelor’s degree. Detailed information is provided in Table 3.

**Table 3:** Characteristics of ICU nurses.

Item	Group	Number of people (cases)	Proportion (%)
Gender			
	Male	10	21.3
	Female	37	78.7
Age			
	25 years and below	2	4.3
	26–30 years	18	38.3
	31–35 years	14	29.8
	36–40 years	13	27.7
Education level			
	Associate degree	5	10.6
	Bachelor’s degree	42	89.4
Title			
	Nurse	3	6.4
	Registered nurse	42	89.4
	Head nurse	2	4.3
Years of experience in ICU			
	1–5 years	12	25.5
	6–10 years	13	27.7
	11–15 years	16	34
	16–20 years	5	10.6
	Over 20 years	1	2.1

Delirium knowledge was measured with 27 items questionnaire with total scores 0–27. Average total knowledge score increased significantly from 17.00 (IQR 16.00–18.00) at baseline to 25.00 (IQR 24.00–27.00) 3 months after training. A Wilcoxon signed-rank test was paired with a statistically significant improvement (Z = −5.978, *p* < 0.001) and large effect size (*r* = 0.87). These results suggest that TTT program improved knowledge of delirium knowledge of ICU nurses.

### 3.2 Behavioral-level evaluation results

We conducted practical assessments of delirium evaluation skills using 5 standard cases. We compare mastery of delirium assessment skills before training and after training in Table 4. The nurses knew well Case 2 before training and after training (*p* = 0.707), but did not master all other cases (all *p* < 0.05). Effect size for significant case comparisons is: Case 1, *r* = 0.46; Case 3, *r* = 0.48; Case 4, *r* = 0.57; Case 5, *r* = 0.46. Total accuracy score (i.e., sum of the 5 cases) (range 0–50) increased from 44 (IQR 40–46) baseline to 48 (IQR 47–50) post training, with large effect size (*Z* = 4.428, *r* = 0.65, *p* < 0.001).

**Table 4:** Comparison of ICU nurses’ scores on delirium assessment practice before and after training [score, M(Q1, Q3)].

Item	Baseline score (median, IQR)	3 Months post-training (median, IQR)	*Z* value	*P* value
Case 1	10 (10,10)	10 (10,10)	3.162	0.002
Case 2	10 (8,10)	10 (8,10)	0.375	0.707
Case 3	8 (6,10)	10 (10,10)	3.303	<0.001
Case 4	8 (8,10)	10 (10,10)	3.883	<0.001
Case 5	8 (8,10)	10 (8,10)	3.170	0.002
Overall	44 (40,46)	48 (47,50)	4.428	<0.001

Each case was scored on a scale of 0 to 10 based on five assessment components, with each component worth 2 points. The total score is the sum of the five case scores, ranging from 0 to 50.

### 3.3 Results-level evaluation results

Self-efficacy improvement was observed for ICU nurses, with pre-training scores (median = 30, IQR [26,31]) significantly larger than post-training scores (median = 36, IQR [31,40]), using Wilcoxon signed-rank test (*Z* = –4.094, *p* < 0.001) and large effect size (*r* = 0.60). This suggests training model improved self-efficacy for ICU nurses.

### 3.4 Qualitative findings: thematic analysis of nurses’ experiences

Semi-structured interviews with 12 nurses and one focus group (three participants) revealed four major themes and several sub-themes that elucidate the mechanisms and outcomes of the TTT program.

#### 3.4.1 Theme 1: experiential learning trilogy—simulation, practice, and teaching

Nurses consistently identified the standardized patient simulation as the most impactful component of the training. This hands-on experience transformed abstract knowledge into embodied understanding. “The simulation training was the most helpful. You could experience it firsthand… being able to assess different types of patients on the spot and combine the examples to deepen my impression.” (Nurse 2) “Simulation gives you a complete process, with the language environment and the setting. It’s like an exam—you remember it deeply” (Nurse 6).

Repeated clinical application was described as essential for internalizing skills: “Practice makes perfect. I assess every patient… it’s become a work habit, like taking vital signs.” (Nurse 3) “The more you assess, the more you encounter different presentations, and you become more skilled at applying the tool in various situations.” (Nurse 1).

The act of teaching peers deepened understanding and revealed knowledge gaps: “When you teach others, you learn it again yourself. Questions from others make you realize things you had not thought about.” (Nurse 7) “If someone asks me, I might need to go back and review the teaching videos or case materials. Teaching forces you to clarify your thinking.” (Nurse 9).

#### 3.4.2 Theme 2: from experience to evidence—the construction of confidence

Nurses attributed their increased confidence to three interconnected sources: objective tools, positive feedback, and systematic knowledge.

Objective tools as anchors for certainty: “Before, everyone had their own subjective judgment. Now we have this scale—it’s a unified standard, more convincing.” (Nurse 4) “Because you have a standard, you know your assessment is based on criteria. It gives you evidence.” (Nurse 1).

Reinforcement through successful application: “After each assessment, when I report to the doctor and the patient is diagnosed with delirium, and treatment helps—that builds confidence.” (Nurse 2).

Systematic knowledge as a cognitive framework: “The concepts are clearer now. The measuring stick for assessment is more precise.” (Nurse 5) “Before, it was vague and general. After training, you have a systematic understanding—you know where each feature fits.” (Nurse 10).

#### 3.4.3 Theme 3: environmental forces—barriers and facilitators in clinical application

Neurological deficits posed significant challenges: “Intubated patients, those on sedatives, or with fluctuating consciousness—these are hard to assess accurately.” (Nurse 3) “Patients with hearing or vision problems, or limb weakness—you have to adapt, but it’s difficult.” (Nurse 8).

“The biggest difficulty is defining the RASS score when patients are on sedatives… you might need another person to help you decide.” (Nurse 1).

Patient non-cooperation was another common obstacle: “He does not want to cooperate anymore. You assess every shift, every day—eventually he gets tired of it.” (Nurse 11) “Sometimes they just do not want to talk to you, and you cannot tell if they are really confused or just unwilling.” (Nurse 9).

Peer support emerged as a key enabling resource: “When I’m not sure, I’ll discuss with my colleagues or the team leader. We’ll assess together and compare.” (Nurse 12) “We communicate during shift handovers. If there’s disagreement, we’ll discuss and come to a consensus.” (Nurse 4).

Timing dilemmas also surfaced: “Should we assess at a fixed time, or whenever needed? What’s the best time of day? If you wake a patient at night to ask questions, they might not answer clearly.” (Nurse 6).

#### 3.4.4 Theme 4: systemic spillover effects—from individual learning to team impact

Enhanced nurse-physician communication: “Now when I talk to doctors, I have evidence. They’re more convinced.” (Nurse 4) “You can tell the doctor, ‘We assessed using the tool, and here are the results.’ Then the doctor might adjust treatment accordingly.” (Nurse 1).

From reporter to active participant in clinical decisions: “I’ll follow up on whether the doctor prescribed medication or other interventions. Before, I just reported and stopped there.” (Nurse 2) “We can discuss with the doctor: maybe reduce sedation, or consider non-pharmacological interventions.” (Nurse 7).

Improved recognition of hypoactive delirium: “We’re screening out hypoactive delirium cases now. Before, we ignored them—we only thought of hyperactive delirium.” (Nurse 5).

Although the majority of participants supported the effectiveness of the TTT model, several nurses observed that the quality of peer teaching might fluctuate based on the trainer’s experience. This variability is reflected in the recommendations from participants for standardized video resources (see Emerging Insights).

#### 3.4.5 Emerging insights: nurses’ recommendations for optimizing future training

Participants offered concrete suggestions to improve the TTT model, with standardized video resources being the most frequently mentioned: “Record standardized teaching videos… so everyone learns from the same source. When different people teach, they might emphasize different details.” (Nurse 2) “If we had videos of the five case types—let nurses see how to assess different patients—that would be more concrete than just explaining the form.” (Nurse 5) “A video would allow us to review when we encounter problems. We could watch it repeatedly and discuss with colleagues.” (Nurse 4).

Other suggestions included flipped classroom approaches and information technology: “Small groups each take a case and teach it. The instructor just comments—that’s a good way to learn.” (Nurse 5) “In the future, maybe AI or facial expression capture could help. Current tools have limitations, especially with non-cooperative patients.” (Nurse 8).

## 4 Discussion

### 4.1 The TTT program addresses real-world ICU training challenges

Delirium has a high incidence in ICU patients and is an independent risk factor for increased ICU mortality and prolonged hospitalization (17). Delirium can bring a series of serious consequences to patients and affect their prognosis (18). Therefore, to ensure its early detection, routine assessment for delirium is a trend in critically ill patient’s care; however, implementation of mandatory screening without standardized training may lead to inaccurate delirium assessment results. Although researchers have adopted a variety of methods, such as remote training (12) and case-based training (11), for the training of nurses on delirium in recent years, studies still show that nurses have problems in clinical delirium assessment, such as inaccurate assessment and a perception that assessment tools are complex (8–10). The reason may be that the training method lacks practical operation guidance, is mostly one-to-many, and is not very targeted. In addition, the training coverage, which is affected by different shifts in the ICU, is not wide enough, resulting in a decline in training effectiveness. Our qualitative findings (Theme 3) substantiate these challenges, revealing that time constraints and patient non-cooperation remain persistent barriers, but also identify the peer-support network fostered by TTT as a novel, internally generated solution.

In this study, we applied a TTT program to delirium training in the hyperbaric ICU environment to provide empirical support for innovation in training programs in this field.

The advantage of the TTT program is that it relies on training instructors to provide training. After the trainers complete the training, they can conduct secondary training to train other nurses in the form of a pyramid to learn delirium knowledge and skills, so that more people can receive training, which can alleviate the problem of insufficient training teachers. At the same time, trainers can conduct training in different shifts to alleviate the impact of ICU shifts. The TTT program is a highly cost-effective educational model. Previous studies have shown that the TTT program exhibits superior effectiveness compared with that of traditional lecture-based training (19, 20).

### 4.2 Advantages of the train-the-trainer program in training ICU nurses

The Kirkpatrick model provided a structured framework for multi-level evaluation, while the embedded qualitative component illuminated the mechanisms underlying the quantitative changes. Integrating these two sources of evidence yields a comprehensive understanding of how the TTT program achieved its effects—not merely that knowledge, skills, and self-efficacy improved, but how and why these improvements occurred.

At learning level, participants had significantly higher delirium knowledge scores after training (*p* < 0.05), especially in regards to tools. Qualitative results revealed this knowledge improvement through simulation practice and peer teaching. Nurses reported that hands-on simulation transformed abstract knowledge into tangible understanding, while teaching peers made them develop and articulate their knowledge. This observation is consistent with Experiential Learning Theory, which states that concrete experiences and active experimentation are essential for deep learning. By combining both simulation and peer teaching, TTT nurses passed from mere memorization to a whole conceptual understanding.

At the behavioral level, quantitative assessment using standardized cases revealed significant improvement in clinical accuracy (*p* < 0.001). The qualitative data elucidated this change through the mechanism of repeated application. Nurses described how sustained clinical practice transformed deliberate effort into habitual competence, with assessment becoming “like taking vital signs.” This progression from conscious application to automatic integration into daily practice represents the ultimate goal of clinical education. The TTT program’s emphasis on sustained application—reinforced by peer accountability—enabled this transition (21).

At the results level, the dramatic increase in self-efficacy (*p* < 0.001) finds comprehensive explanation through Social Cognitive Theory (22), which identifies four sources of self-efficacy. Qualitative findings demonstrated that the TTT program activated all four sources: mastery experiences through successful assessments confirmed by patient outcomes; vicarious experiences through observing peers; social persuasion through collegial consultation and support; and reduced emotional arousal through systematic training. The convergence of all four sources explains the substantial and sustained self-efficacy gain observed quantitatively.

Beyond individual learning, two additional themes emerged with significant implications for practice. First, contextual barriers-particularly neurological deficits and patient non-cooperation-highlight limitations of current assessment tools in specialized settings. Importantly, the peer support network that emerged organically represents an unanticipated but valuable resource for sustaining learning. Second, systemic spillover effects extended beyond individual competence to influence interprofessional collaboration and patient care quality. Enhanced nurse-physician communication and improved recognition of hypoactive delirium represent meaningful contributions that quantitative measures alone could not capture.

A particularly novel finding was nurses’ repeated call for standardized video resources, reflecting an inherent tension in the TTT model: while peer-to-peer training increases reach and efficiency, it may introduce variability in knowledge transmission. This suggests that future iterations should consider supplementing peer training with standardized multimedia resources to ensure consistency while maintaining the benefits of peer learning. This aligns with the concept of blended learning in medical education (23), which combines the scalability of digital resources with the interpersonal benefits of face-to-face instruction.

The qualitative results summarize Bandura’s four sources of self-efficacy: (1) Mastery experiences: Nurses reported that after each assessment, when I report to the doctor and the patient is diagnosed with delirium and treatment helps, then they were confident. This direct experience of success is the strongest source of self-efficacy. (2) Vicarious experiences: Nurses observed peers during simulation training. It allowed nurses to learn from successes and mistakes of others without risking themselves. One nurse said: “When colleagues do it correctly, I know I can do it”. (3) Social persuasion: Peer support and discussions (e.g., “When I’m not sure, I’ll discuss with my colleagues”) encouraged nurses to believe in themselves. (4) Physiological and emotional states: Structured training relieved nurses’ anxiety for delirium assessment and made “vague and general” into a systematic understanding. TTT engaged all four sources simultaneously. The authors summarized quantitatively significant self-efficacy gains observed quantitatively.

### 4.3 Limitations

Although the evaluation is based on the Kirkpatrick model, we have some limitations. Small sample size and single institution settings may limit generalization. Future studies should try to test results with larger sample sizes across multiple institutions and investigate the applicability and long-term effects of the TTT program in different contexts. Although the qualitative component contributed to our analysis, single-center design may limit transferability. The qualitative information may be rich but only from the perspective of a small number of nurses. Future multi-site qualitative studies could account for more variations in context. Video resources requested from participants should also be evaluated. The lack of a control group in this quasi-experimental design is also a major limitation. Without a comparator group, confounding effects such as time effects, maturation effects, testing effects cannot fully account for confounding effects such as time effects, maturation effects etc. Future studies should consider adding a control group or using other designs such as regression discontinuity to enhance causal inference. We did not perform a formal *a priori* sample size calculation. The sample of 47 nurses represented the entire eligible nursing staff of the study ICU which may limit power to detect smaller effect sizes. Future studies should include formal power analyses to ensure adequate sample sizes.

We evaluated 3 levels of the Kirkpatrick model (learning, behavior, and results) as performed by self-efficacy, but not patient level outcomes (delirium duration, time spent in the ICU, or mortality) as results at organizational level. Follow-up should include these higher levels of results to evaluate the impact of TTT programs on quality of care.

## 5 Conclusion

This mixed-methods study demonstrates that the train-the-trainer (TTT) program significantly improves ICU nurses’ knowledge, clinical assessment skills, and self-efficacy in delirium care. The qualitative findings elucidate the mechanisms underlying these improvements: simulation-based practice transforms abstract knowledge into embodied competence; repeated clinical application builds habitual expertise; and teaching others deepens understanding while fostering peer support networks.

Beyond individual learning, the TTT program generated systemic benefits including enhanced nurse-physician communication, improved recognition of hypoactive delirium, and the emergence of collaborative peer support systems. However, contextual barriers—particularly the challenges posed by neurological deficits and patient non-cooperation—highlight the limitations of current assessment tools in specialized settings.

A novel finding with important implications for practice was nurses’ call for standardized video resources to supplement peer training, addressing the challenge of knowledge consistency in pyramid-style dissemination models. This suggests that future TTT programs should consider integrating blended learning approaches that combine the scalability of peer training with the consistency of standardized multimedia resources.

## Data Availability

The raw data supporting the conclusions of this article will be made available by the corresponding author upon reasonable request.
